# Engineering Protein-Based HIV Entry Inhibitors: Advances, Challenges, and Translational Strategies

**DOI:** 10.3390/biom16091221

**Published:** 2026-08-22

**Authors:** Rashmi Kumariya, Carole A. Bewley

**Affiliations:** Natural Products Chemistry Section, Laboratory of Bioorganic Chemistry, National Institute of Diabetes and Digestive and Kidney Diseases, National Institutes of Health, Bethesda, MD 20892, USA; caroleb@intra.niddk.nih.gov

**Keywords:** HIV entry inhibitors, nanobodies, lectins, HIV neutralization, pharmacokinetics, Fc-mediated immune effector functions, manufacturability

## Abstract

Human immunodeficiency virus (HIV) is an enveloped virus with a remarkable capacity for genetic diversification, enabling rapid escape from host immune responses and therapeutic interventions. Despite extensive global efforts, the development of an effective vaccine has remained elusive owing to the virus’s high genetic variability and antigenic diversity. Consequently, considerable effort has been directed toward the development of therapeutic agents targeting viral entry, reverse transcriptase, integrase, protease, and more recently, capsid. Although antiretroviral therapy (ART) remains the cornerstone of HIV treatment, it is associated with challenges including drug resistance, adverse side effects, and limitations in access and affordability. Targeting viral entry offers distinct advantages by blocking infection at the earliest stage of the viral life cycle and enabling the neutralization of free virions, as well as Fc-mediated elimination of HIV-infected cells in some cases. This review highlights promising protein-based HIV entry inhibitors that have demonstrated efficacy in preclinical studies, and discusses ongoing efforts to optimize their valency, avidity, specificity, serum half-life, effector functions, and production platforms to improve their therapeutic potential and economic feasibility.

## 1. Introduction

Despite remarkable advances in HIV prevention and treatment, HIV-1 continues to pose a significant global public health challenge, with nearly 40 million people currently living with the virus worldwide [1]. Although an estimated 1.3 million individuals acquired HIV in 2024, representing a 40% reduction in new infections since 2010 and a 61% decline from the epidemic peak in 1995, the global burden of HIV remains substantial [2]. Over the past four decades, scientific breakthroughs have transformed HIV infection from a fatal disease into a manageable chronic condition, with antiretroviral therapy (ART) playing a pivotal role in reducing HIV-associated morbidity and mortality [2]. Nevertheless, the need for lifelong treatment, the emergence of drug resistance, treatment-related toxicities, and the persistence of latent viral reservoirs continue to impede efforts toward achieving a definitive cure. These ongoing challenges have fueled the development of innovative therapeutic strategies targeting multiple stages of the HIV life cycle. To date, the Food and Drug Administration (FDA) has approved numerous drugs for the treatment of HIV, the majority of which fall under ART, a regimen that combines two or more agents to effectively suppress HIV infection. These FDA-approved drugs are classified into nine major categories based on their mechanisms of action: nucleoside reverse transcriptase inhibitors (NRTIs), non-nucleoside reverse transcriptase inhibitors (NNRTIs), integrase strand transfer inhibitors (INSTIs), protease inhibitors (PIs), fusion inhibitors (FIs), CCR5 antagonists, attachment inhibitors, post-attachment inhibitors, and capsid inhibitors [3]. Beyond these, the FDA recognizes two additional categories: pharmacokinetic enhancers, which boost the efficacy of existing anti-HIV agents, and fixed-dose combination medicines, which consolidate two or more antiretrovirals into a single formulation for improved anti-HIV efficacy and patient convenience [3]. The majority of these drug classes target HIV replication and collectively contribute approximately 20 agents to the therapeutic arsenal, while those targeting HIV entry represent a comparatively smaller subset of only four approved drugs (Table 1). Across all categories, most approved agents are small molecules that despite their ability to penetrate HIV-infected cells and effectively suppress viral replication, are hampered by short half-lives and thus the need for frequent dosing. These pharmacokinetic limitations pose a significant challenge to patient adherence, a cornerstone of successful HIV management. In contrast, when the therapeutic goal shifts from inhibiting HIV replication to blocking HIV entry, protein-based therapeutics are emerging as a particularly compelling alternative. Unlike small molecules that must permeate host cells to exert their effect, entry inhibitors act extracellularly, a mechanism ideally suited to larger biologics. Proteins, by nature, offer superior pharmacokinetic profiles, including extended half-lives and less frequent dosing requirements, which translate directly into improved patient compliance. In this light, HIV entry inhibition represents not only a mechanistically distinct but also a strategically promising avenue, one that could meaningfully address both the virological and patient adherence challenges central to combating the global HIV epidemic.

**Figure 1:**
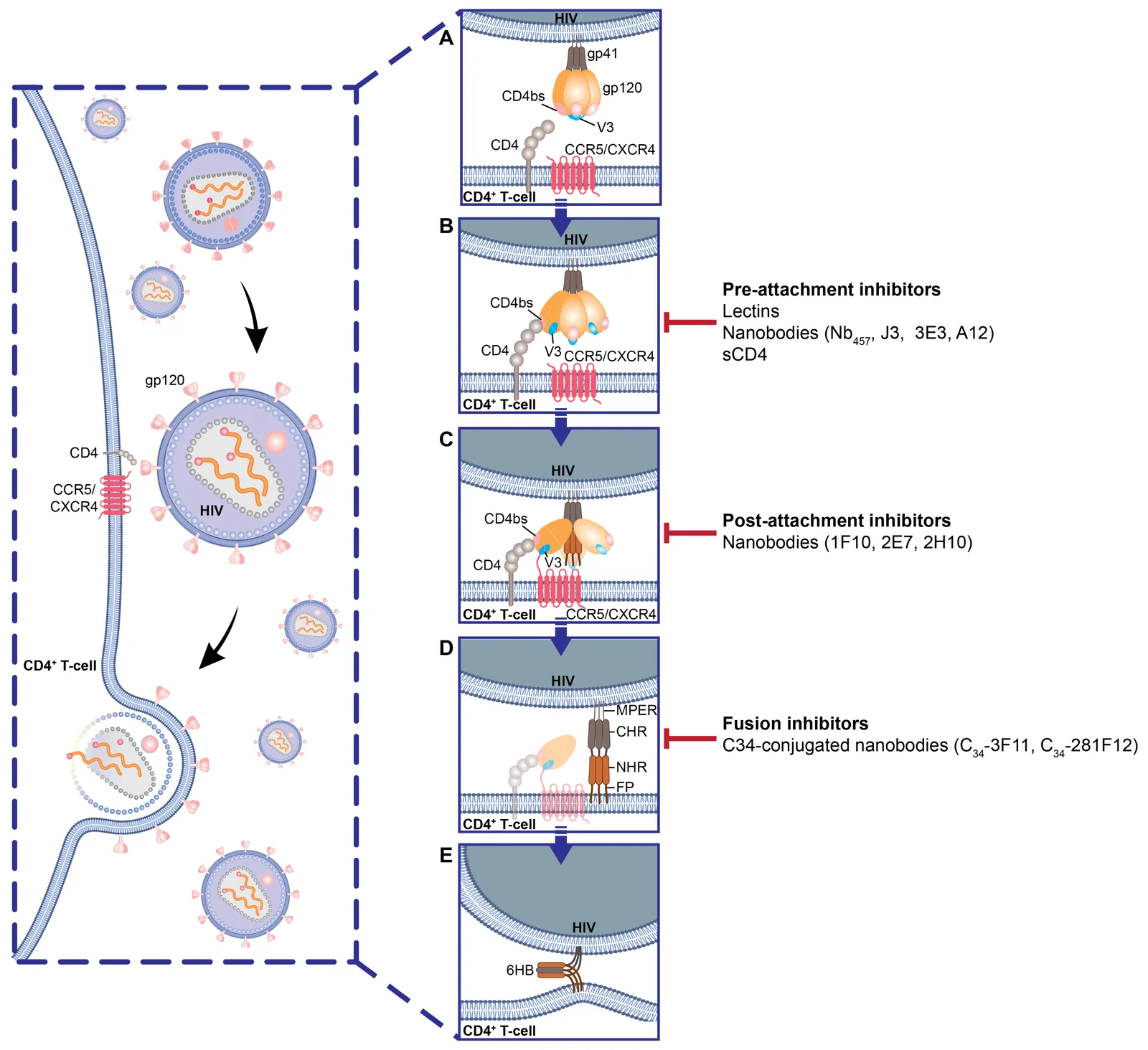
HIV entry process and inhibitors. HIV-1 initiates infection by engaging the CD4 receptor on the surface of CD4^+^ T cells through its envelope glycoprotein gp120 (**A**). Binding of gp120 to CD4 triggers a series of conformational changes that expose the V3 loop (**B**), enabling subsequent interaction with the co-receptors CCR5 or CXCR4 (**C**). These interactions bring the viral and cellular membranes into close proximity and activate gp41-mediated fusion, leading to insertion of the fusion peptide into the host cell membrane (**D**) and ultimately the fusion of viral and cellular membranes (**E**). Shown are representative protein-based HIV entry inhibitors, including nanobodies, lectins, soluble proteins, and peptide-conjugated nanobodies, which interfere with distinct stages of the HIV entry process as indicated by red blunt-ended arrows.

HIV-1 entry into host cells is initiated when the viral envelope glycoprotein gp120 binds to the host CD4 receptor, inducing conformational changes that facilitate subsequent interaction with one of the host co-receptors, CCR5 or CXCR4. This interaction activates the gp41 subunit, promoting fusion between the HIV-1 and host cell membranes and ultimately enabling viral entry [4]. Numerous biomolecules targeting HIV-1 entry have been identified to date, including small molecules, peptides, nanobodies, and lectins. These agents inhibit viral entry by targeting key components of the entry process, such as host receptors or co-receptors, the HIV-1 gp120 or gp41 proteins, or intermediate pre-attachment, post-attachment, fusion complexes (Figure 1). Several small-molecule and peptide-based inhibitors have advanced to clinical use and received FDA approval for both prophylactic and therapeutic applications [5,6,7] (Table 1). Ongoing efforts continue to enhance their efficacy through innovative strategies, including peptide conjugation using orthogonal chemical and supramolecular approaches to improve potency [8], fusion with nanobodies to enhance target specificity [9], and attachment to albumin- or Fc-binding motifs to extend half-life and therapeutic effectiveness [10]. As this review primarily focuses on protein-based HIV-1 entry inhibitors, the following sections highlight recent advances and emerging developments in this rapidly evolving field and the strategies discovered to improve their efficacy, pharmacokinetics, and manufacturability.

## 2. Protein-Based HIV-1 Entry Inhibitors

The full-length antibodies are among the most effective and safest HIV-1 entry inhibitors. Beyond direct HIV-1 neutralization, antibodies can also activate immune effector functions to eliminate HIV-1 infected cells. Several anti-HIV broadly neutralizing antibodies (bnAbs) are in clinical trials for the management of HIV infection, but none are yet approved by the FDA [11]. The comprehensive perspectives on the therapeutic potential and clinical advancement of anti-HIV bnAbs has been extensively covered in recent reviews [11,12]. Though anti-HIV bnAbs have shown promising therapeutic potential, their large molecular weights complicate their production, thereby increasing production costs. These limitations have driven the development of alternative smaller molecular-weight protein-based inhibitors that retain potent anti-HIV activity and desirable pharmacokinetics that in turn can lower manufacturing costs. Several low molecular-weight protein-based inhibitors have demonstrated considerable potential as next-generation HIV-1 entry inhibitors such as soluble host proteins, Fabs, nanobodies, and lectins (Table 2). Soluble host proteins are derived from endogenous host factors and typically represent components of the innate immune system or soluble forms of host proteins targeted by HIV [13], while Fabs comprise the antigen-binding fragments of anti-HIV bnAbs, retaining their target specificity in a smaller format [14]. Nanobodies represent the smallest naturally occurring antibody-derived antigen-binding domains, combining high specificity with improved accessibility to sterically restricted epitopes [15]. Lectins are carbohydrate-binding proteins that can exploit multivalent interactions with the densely glycosylated HIV envelope, enabling potent recognition and inhibition of HIV entry [16]. Selected examples of these promising protein scaffolds are discussed below.

### 2.1. Soluble Host Proteins

Several soluble host proteins represented by soluble receptors or components of innate immunity have been shown to prevent HIV-1 entry. Examples include soluble CD4 (sCD4), α- and β-defensins, lactoferrin, C1q protein, and ligands that bind either the CD4 receptor or coreceptors CCR5 or CXCR4 [17,18,19]. Unlike other host proteins, sCD4 is HIV specific and has been extensively studied because it specifically targets the highly conserved CD4-binding site (CD4bs) on gp120 proteins and HIV-1 cannot escape sCD4 without a fitness cost [20]. It inactivates HIV-1 by triggering a transient intermediate state of gp120 prematurely, thus inhibiting HIV-1 infection [21]. Because it is a truncated form of the membrane anchored CD4 receptor, it acts as a competitive decoy receptor binding to gp120 and inhibiting further interactions with the host CD4 receptor [22]. Since all HIV-1 isolates require CD4 receptors to initiate infection, sCD4-based neutralization is independent of HIV-1 tropism i.e., it can neutralize CCR5-tropic, CXCR4-tropic, and dual-tropic isolates [23]. Haim et al. showed that sCD4 inhibited HIV infection at nanomolar concentration in CD4^+^CCR5^+^ CfTH2 cells, however, in CD4^−^CCR5^+^ CfTH2 cells, sCD4 surprisingly enhanced the HIV infectivity perhaps by promoting HIV attachment [21]. sCD4 has been engineered in many ways to improve its functionality and stability (Table 2) [24,25,26]. Also, binding of sCD4 to HIV-1 gp120 induces conformational changes in gp120/gp41, exposing the hidden epitopes on gp41 to make them accessible to anti-gp41 antibodies and peptides [27,28]. This mechanism results in better HIV-1 neutralization profile of conjugates like sCD4-FI_T45_ than sCD4 itself [26]. Thus, sCD4 has mostly been explored as a combination therapy or as a fusion protein with other active anti-HIV candidates [26,27,28].

**Table 2:** Protein-based HIV entry inhibitors with proven in vitro anti-HIV efficacy.

Proteins	Engineered	Target	Valency ^a^	Specificity	In Vitro Efficacy				References
					Cells	HIV Strains	IC_50_	Breadth ^b^	
**Soluble proteins**									
sCD4	-	CD4b (HIV-1 gp120)	Monovalent	Monospecific	CD4^+^ CCR5^+^ Cf2Th	AD8	4.0 µg/mL	-	[21]
						YU2	0.6 µg/mL		
					TZM-Bl	JRFL	2.0 µg/mL	BN	[26]
						BaL01	0.025 µg/mL		
	sCD4-FI_T45_	CD4b (HIV-1 gp120) and NHR (gp41)	Monovalent	Bispecific	TZM-Bl	JRFL	0.016 µg/mL	BN	[26]
						BAL01	0.006 µg/mL		
	CD4-Ig	CD4b (HIV-1 gp120)	Bivalent	Monospecific	TZM-Bl	YU2	0.293 µg/mL	BN	[29]
	eCD4-Ig	CD4b and coRb (HIV-1 gp120)	Bivalent	Bispecific			0.030 µg/mL		
**Fabs**									
b12_Fab_	-	CD4b (HIV-1 gp120)	Monovalent	Monospecific	C8166	IIIB	0.13 µg/mL	-	[30]
**Nanobodies**									
Nb_457_	Nb_457_-Nb_HSA_-Nb_457_	D1 and D2 domains of CD4 receptor and HSA	Bivalent	Monospecific	GHOST(3)-X4R5	BAL	0.359 µg/mL	BN	[31]
	Nb_457_-Fc	D1 and D2 domains of CD4 receptor and Fc receptors	Bivalent	Monospecific			0.18 µg/mL	BN	
J3	-	CD4b (HIV-1 gp120)	Monovalent	Monospecific	TZM-Bl	398F1	33 nM	BN	[32]
	J3-J3		Bivalent				9 nM		
	J3-J3-J3		Trivalent				12 nM		
	J3-Fc		Bivalent				3 nM		
	J3-1F10	CD4b and V3 loop (HIV-1 gp120)	Monovalent	Bispecific			9 nM		
	J3-1F10-Fc						6 nM		
	J3-2E7	CD4b (HIV-1 gp120) and HR1 (HIV-1 gp41)					57 nM		
	J3-2E7-Fc	CD4b (HIV-1 gp120) and HR1 (HIV-1 gp41)					11 nM		
3E3	-	CD4b (HIV-1 gp120)	Monovalent	Monospecific	TZM-Bl	BAL26	0.104 µg/mL	BN	[33]
A12	-	CD4b (HIV-1 gp120)	Monovalent	Monospecific	TZM-Bl	YU2	34 µg/mL	BN	[34]
						BAL	3.6 µg/mL		
1F10	-	V3 loop (HIV-1 gp120)	Monovalent	Monospecific	TZM-Bl	398F1	0.81 µM	BN	[32]
	1F10-1F10		Bivalent				0.78 µM		
	1F10-Fc		Bivalent				1.4 µM		
2E7	-	HR1 (HIV-1 gp41)	Monovalent	Monospecific	TZM-Bl	398F1	1.28 µM	BN	[32]
	2E7-2E7		Bivalent				1.2 µM		
	2E7-Fc		Bivalent				0.94 µM		
2H10	-	MPER (HIV-1 gp41)	Monovalent	Monospecific	TZM-Bl	BAL26	>50 µg/mL	-	[35]
	2H10-F		Monovalent				19.38 µg/mL	BN	
	2H10-RKRF		Monovalent				9.63 µg/mL		
	Bi-2H10		Bivalent				6.05 µg/mL		
3F11	3F11-C34	Host CD4 receptor	Monovalent	Bispecific	TZM-Bl	YU2	4.2 pM	BN	[9]
CD4b1	CD4b1-C34	Host CD4 receptor	Monovalent	Bispecific			12 pM		
281F12	281F12-C34	Host CXCR4 co-receptor	Monovalent	Bispecific			170 pM		
**Lectins ^c^**									
GRFT	-	Mannose-rich glycans (HIV-1 gp120)	Bivalent	Monospecific	TZM-Bl	YU2	2.4 pM	BN	[36]
	mGRFT-Fc		Bivalent	Monospecific			28.6 pM		
CV-N	-	Mannose-rich glycans (HIV-1 gp120)	Monovalent	Monospecific	TZM-Bl	SC422661.8	4.4 nM	BN	[37,38,39]
	CV-N_2_L0		Bivalent	Monospecific			0.49 nM		
	CV-N_3_L0		Trivalent	Monospecific			0.38 nM		
AH	-	Mannose-rich glycans (HIV-1 gp120)	Monovalent	Monospecific	HOS-CD4-CCR5^+^	QH0692.42	60.42 nM	BN	[40]
	Avaren-Fc		Bivalent	Monospecific			5.61 nM		
	Avaren- VRC01_Fab_	Mannose-rich glycans and CD4B (HIV-1 gp120)	Bivalent	Monospecific	HOS-CD4-CCR5^+^	QH0692.42	0.17 nM	-	[41]
BanLec	-	Mannose-rich glycans (HIV-1 gp120)	Bivalent	Monospecific	TZM-Bl	SVPB5	0.3 nM	-	[42]
	-		Bivalent	Monospecific	TZM-Bl	BAL	1.1 nM	-	[43]
	BanLec Y46K		Monovalent				2.9 nM	-	
MVN	-	Mannose-rich glycans (HIV-1 gp120)	Monovalent	Monospecific	U87.CD4.CCR5	BAL	22 nM	BN	[44]

^a^ Valency, defined as number of epitope-recognizing subunits per molecule. ^b^ BN: Broadly neutralizing candidates, candidates capable of neutralizing a diverse panel of HIV strains representing multiple genetic clades. ^c^ Lectins’ valency: Note that the valency listed in the Table corresponds to the number of epitope-recognizing subunits as in (a). However, each lectin may have multiple individual carbohydrate binding sites per subunit.

### 2.2. Fabs

Fab fragments derived from the antigen-binding domain of full-length antibodies offer several advantages. Due to their small size, Fabs show better tissue penetration and access to cryptic epitopes, lower non-specificity and immunogenicity, and easier production than full-length antibodies; however, they cannot elicit Fc-mediated immune effector responses [14]. Some Fab fragments derived from bnAbs were shown to retain gp120-binding ability and HIV-1 neutralization activity, including 2G12_Fab_ and b12_Fab_ [30,45,46]. Though b12_Fab_ showed 10-fold reduced HIV neutralization efficacy as compared to b12-IgG, the ability of b12_Fab_ to retain HIV neutralization in nanomolar range suggests that bivalency and hence multivalent interaction is dispensable for b12 IgG [30]. Also, it is interesting to note that b12 _Fab_ showed inhibition post-entry in HIV-1 infection, while b12-IgG1 inhibits the primary fusion-uncoating mechanism [30]. So, the same Fab region may act via a different mechanism when separated from its parent IgG1. For other Fabs, such as 2G12_Fab_, the removal of the domain-exchanged IgG architecture required for multivalent interactions compromised their anti-HIV efficacy [45,46]. Hence Fabs are mainly explored to gain structural insights into interactions between HIV-1 gp120 and bnAbs rather than being explored as therapeutics [47,48].

### 2.3. Nanobodies

Nanobodies have recently emerged as versatile and promising therapeutic agents. They are unique antibody fragments characterized by their small molecular weight (~15 kDa) coupled with retention of high affinity and specificity typically associated with conventional antibodies [15]. Derived from the variable domain of heavy-chain-only antibodies found in camelids, nanobodies possess exceptional biophysical properties that enable them to access cryptic or sterically restricted epitopes often inaccessible to full-length antibodies [15]. These advantages have attracted considerable interest in their application across diagnostics, imaging, and therapeutic development. Several nanobodies have demonstrated potent anti-HIV activity by either targeting host receptors or HIV-1 gp120 or gp41 proteins functioning as pre-attachment and post-attachment entry inhibitors, CCR5 antagonists, or fusion inhibitors (Table 2).

Nb_457_, a potent anti-CD4 nanobody isolated from a CD4-immunized alpaca, exhibits broad-spectrum HIV-1 inhibitory activity at the pre-attachment stage [31,49]. Its anti-HIV effect is mediated through binding-induced conformational changes in the CD4 receptor that hinder viral entry [31]. To enhance its pharmacokinetic properties, Nb_457_ has been engineered into Fc-fusion and heterotrimeric albumin-binding (Nb_457_-Nb_HSA_-Nb_457_) formats [31,49]. Notably, the trimeric construct achieved complete HIV neutralization in humanized mouse models, whereas the Fc-fusion variant demonstrated substantial antiviral activity but did not achieve complete protection, even at high doses [31]. Several nanobodies, including J3, 3E3, A12, JM, and B9, target the highly conserved CD4-binding site on the HIV-1 gp120 envelope glycoprotein [50]. Among these, J3 is the most extensively characterized and exhibits exceptional neutralization breadth and potency [32]. Structural studies have shown that J3 mimics binding by the CD4 receptor to the prefusion-closed trimer for the primary site of CD4 recognition as well as a secondary quaternary site [51]. Engineering J3 into bivalent, trivalent, and Fc-fused formats has further enhanced its neutralizing activity compared with the monovalent form [32]. Similarly, 3E3, A12, JM, and B9 have demonstrated broad and potent HIV-1 neutralization profiles [34,52,53]. Beyond antiviral activity, J3, 3E3, and B9 have been shown to selectively target gp120-expressing tumors with minimal background signal, where they were labeled with ^18^F for imaging gp120-expressing tumors [54]. Despite exhibiting ultra-potent and HIV-specific activity in vitro [32,33,52], a few in vivo studies of these candidates have been conducted thus far leaves open their potential as anti-HIV candidates.

Additional nanobodies targeting conserved epitopes on the HIV-1 gp120 envelope glycoprotein have also been reported. For example, 1F10 binds the HR1 on the V3 loop [55], 2H10 binds MPER [56], and 2E7 binds HR1 on gp41 [55]. However, the antiviral potency of these nanobodies is generally lower than that of nanobodies directed against the CD4-binding site. Consequently, strategies that combine these nanobodies with other antiviral modalities may enhance their therapeutic efficacy and broaden their utility as HIV-1 entry inhibitors [57].

Another promising host-targeted strategy involves CD9, a member of the tetraspanin family implicated in membrane fusion processes [58]. CD9 plays an important role in syncytia formation which eventually leads to viral cell-to-cell transmission and viral egress; thus, blocking CD9 may hamper these processes [58]. Recently, the nanobodies T2C001 and T2C002, generated from a CD9-immunized alpaca, were shown to bind CD9 with low-nanomolar affinity. Although they did not directly block HIV-1 entry, they significantly inhibited virus-induced syncytium formation and reduced viral replication in both T cells and monocyte-derived macrophages by blocking CD9 [58]. These findings highlight the therapeutic potential of targeting host factors to complement existing antiretroviral strategies.

Innovative bioconjugation approaches have further expanded the utility of nanobodies in HIV therapy. Conjugation of the fusion inhibitor peptide C34 to CD4- or CXCR4-targeting nanobodies such as 3F11, CD4b1, and 281F12 enhanced antiviral potency by approximately 10,000-fold through selective delivery of C34 to susceptible CD4^+^ T cells [9]. Such strategies underscore the growing role of nanobody-based platforms in the development of next-generation anti-HIV therapeutics.

Although numerous HIV-targeting nanobodies have been identified, to date only one has progressed to preclinical evaluation (Table 3). Further efforts are required to advance these promising candidates toward clinical development as effective HIV prophylactics and therapeutic agents. There still remain substantial opportunities for discovery of novel anti-HIV-1 nanobodies. Continued efforts to identify, engineer, and comprehensively evaluate such candidates will provide a strong foundation for advancing nanobody-based therapeutics as promising interventions for HIV prevention and treatment.

### 2.4. Lectins

Lectins comprise a large and ubiquitous superfamily of carbohydrate-binding proteins, encompassing thousands of members across all domains of life. Althouh thousands have been identified, only a handful, such as griffithsin (GRFT), cyanovirin N (CV-N), actinohivin (AH), banana lectin (BanLec), concanavalin A (ConA), and microvirin (MVN) have emerged as leading antiviral candidates. The multivalent interactions between these lectins and high mannose glycans present on HIV gp120 lead to sub-nanomolar binding and HIV neutralization potencies [16]. All these lectins act as pre-attachment HIV entry inhibitors (Table 2). Below we have discussed some well-studied and well-characterized lectins that show potential to be developed not only as topical microbicides but as successful prophylactic and therapeutic agents.

GRFT is a lectin derived from the red alga *Griffithsia* sp. [59] having a unique domain-swapped dimeric structure with a β-prism fold [60] with each monomer having three sugar-binding pockets. It is well characterized for its multivalent interactions with mannose-rich glycans present on the surface of HIV gp120 and sub-nanomolar binding affinities [60,61]. This gp120 binding ability of GRFT is well translated into its HIV neutralization efficacy by blocking HIV entry via inhibition of interaction between HIV gp120 and host receptors present on CD4^+^ T-cells [60,61]. This anti-HIV potency of GRFT stems from the dimeric nature and C(3)-symmetric core of GRFT as monomerization of GRFT reduces the anti-HIV potency of GRFT [62] and mutations in the C(3)-symmetric core also affect the GRFT’s anti-HIV potency [63]. The anti-HIV efficacy of GRFT has been proven both in vitro and in vivo in the form of a topical microbicide [64] and prophylactic agent [36] (Table 3). In addition, several studies show that GRFT neither induces cytotoxicity nor has any mitogenic effects [36,65,66], underlining its promise as a prophylactic and therapeutic candidate to tackle HIV infection.

CV-N, a potent anti-HIV lectin isolated from *Nostoc ellipsosporum*, is a 11-kDa monomeric protein with two sugar binding pockets that neutralizes several strains of HIV at low nanomolar concentrations [67] by binding high-mannose oligosaccharides present on HIV gp120 [68,69]. Engineering of CV-N as a dimer (CVN_2_) to increase the carbohydrate binding sites resulted in enhanced HIV neutralization by up to 18-fold compared to CV-N [37]. CV-N does not induce cytotoxicity when tested in vitro [39,67] and in vivo [38,39]. CV-N has been tested for its in vivo anti-HIV efficacy as a topical microbicide in a vaginal challenge model where CV-N-treated female macaques were exposed to a pathogenic chimeric SIV/HIV-1 virus, SHIV89.6P. Administration of CV-N conferred protection by ~80% [39]. Because CV-N shows promising anti-HIV efficacy without any adverse effects, it has a potential to be explored further beyond topical microbicides.

AH, isolated from a culture filtrate of the actinomycete strain K97-0003 (*Longispora albida*), is a monomeric lectin with a molecular weight of 12.5 kDa and characterized by three internal, repeating segments each of which has a sugar-binding pocket [70,71]. Owing to this characteristic, AH shows strong affinity towards high-density high-mannose type glycans via multivalent interactions making it highly specific for gp120, unlike CV-N, which does not discriminate between low-density and high-density high-mannose type glycans and has been reported to bind to some host glycoproteins [71]. AH inhibited the cytopathic effects of HIV-1IIIB in MT-4 cells and prevented syncytium formation in both uninfected T-cell and macrophages [70] and in cocultures of persistently HIV-1-infected HuT-78 cells and uninfected CD4^+^ T lymphocytes. AH further inhibited virus transmission to CD4^+^ T-cells, lacks mitogenic activity and does not result in cytokine or chemokine secretion in peripheral blood mononuclear cells, a promising profile for further development [72].

BanLec is a dimeric protein comprising two identical 15-kDa subunits each with two sugar-binding pockets [73]. It binds high-mannose glycans present on gp120 via bidentate interactions resulting in HIV neutralization [43]. It exists as a tetramer in solution where its sugar-binding sites are well positioned to optimally target glycans on gp120 which together with bidentate binding results in HIV neutralization with augmented avidity [43]. BanLec showed broad anti-HIV activity, neutralizing multiple HIV-1 strains with different tropisms. Its anti-HIV activity compares well other potent lectins such as GRFT [42]. While wild-type BanLec is mitogenic, an engineered BanLec H84T mutant is significantly less mitogenic than wild-type and retains its anti-HIV potency [74].

ConA is a homotetrameric lectin isolated from *Canavalia ensiformis* that binds mannose-rich glycans displayed by HIV gp120 [16]. It showed binding to gp120 proteins from four different HIV strains suggesting its less sensitive to mutations in the glycosylation sites [75]. However, there are reports of ConA-resistant HIV-1 strains [76]. Additionally, ConA has shown mitogenic effects and cytotoxicity to hepatocytes [77], which makes its application as anti-HIV agent uncertain unless being engineered to obviate its cytotoxicity and preserve anti-HIV efficacy.

MVN, a monomeric lectin isolated from the cyanobacterium *Microcystis aeruginosa*, binds high-mannose glycans on HIV gp120 [78]. It exhibits antiviral activity against HIV-1 strains of diverse tropisms, inhibits syncytium formation, and blocks viral transmission between infected and uninfected T-cells [44,79]. Although prolonged exposure can select for MVN-resistant HIV-1 variants, these strains remained susceptible to other glycan-binding lectins such as CV-N [44]. Notably, MVN shows no detectable immunostimulatory activity or cytotoxicity [44], supporting its potential as a candidate for HIV prevention and therapy.

There are some other lectins such as *Galanthus nivalis* agglutinin (GNA), *Microcystis viridis lectin* (MVL), *Oscillatoria agardhii* agglutinin (OAA), scytovirin (SVN), isolated from plants, cyanobacteria, and algae, that possess sub-nanomolar anti-HIV potencies [16], but are yet to be explored further for their safety and use as topical microbicides and preventive and therapeutic applications. While most lectins have immunogenic properties raising the concerns about their safety, being proteinaceous in nature, there is a scope of engineering such potent anti-HIV lectins to make them safe for use while retaining their anti-HIV efficacy.

Overall, each class of protein-based HIV entry inhibitor offers a distinct balance of antiviral activity and properties relevant to therapeutic development. Nanobodies combine potent target recognition with exceptional access to sterically restricted epitopes and efficient production, but their small size can result in rapid systemic clearance [15]. Lectins benefit from multivalent recognition of the densely glycosylated HIV envelope and can exhibit broad antiviral activity, although immunogenicity and off-target glycan interactions remain important concerns [16]. Fabs retain the specificity and neutralization properties of their parental bnAbs in a smaller format but lack Fc-mediated effector functions and generally exhibit shorter serum persistence [14]. Soluble host proteins may offer favorable biocompatibility and low immunogenicity, although their antiviral potency and pharmacokinetic properties vary considerably [13]. Thus, no single platform is universally superior, and future clinical success will likely depend on balancing anti-HIV potency with pharmacokinetics, safety, and feasibility of production through rational protein engineering.

## 3. Strategies to Improve HIV Neutralization Efficacy

In HIV entry inhibition, the strength of target engagement is a key determinant of neutralization potency. Initial target recognition is governed by the intrinsic affinity and specificity of an inhibitor, particularly its paratope, for its cognate epitope. Several bnAbs isolated from HIV-infected individuals exhibit exceptional neutralizing activity and target conserved regions of the viral envelope glycoprotein, including the CD4-binding site, V1/V2 apex, and glycan-rich V3 region [80,81,82,83]. However, bnAbs are relatively rare because their conserved epitopes are often shielded by surrounding glycans, neighboring residues, and the quaternary structure of the envelope trimer [45,84]. Despite these challenges, innovative strategies aimed at eliciting bnAbs continue to advance. Extensive structural studies of gp120 and envelope trimers in complex with neutralizing antibodies have elucidated the molecular determinants of effective HIV neutralization [80,81,82,85,86]. These insights provide a foundation for the rational design of inhibitors that either mimic critical antibody-envelope interactions or directly target vulnerable sites on gp120, thereby enhancing antiviral efficacy [87,88]. Beyond leveraging structural information from inhibitor-target complexes, rational optimization of binding interfaces has emerged as a powerful strategy for improving anti-HIV activity. For example, structure-guided mutagenesis, X-ray crystallography, and molecular dynamics simulations identified the conserved aromatic core of GRFT as a key determinant of its high-affinity glycan binding and potent antiviral activity [63]. Mutations within this aromatic core substantially reduce binding affinity and neutralization potency, underscoring its functional importance [63]. Collectively, these findings reveal a distinctive glycan-recognition motif and provide a framework for the rational engineering of glycan-targeting HIV entry inhibitors.

Once the interaction is optimized, valency becomes an additional and highly influential factor, as increased valency enhances overall binding strength through avidity effects. This principle has been successfully exploited across multiple protein scaffolds. For instance, nanobodies, which are inherently monovalent, have been engineered into multivalent formats to improve avidity and neutralization potency. Among anti-HIV nanobodies, J3 has emerged as one of the most potent described to date and its engineered bivalent, trivalent, and Fc-fusion J3 formats have demonstrated superior HIV neutralization compared with its monovalent form [32]. Similarly, the anti-CD4 nanobody Nb_457_ has been engineered as an Fc fusion and as a trimeric construct incorporating a human serum albumin-binding nanobody (Nb_457_-Nb_HSA_-Nb_457_), with both multivalent formats exhibiting enhanced HIV neutralization relative to monovalent Nb_457_ [31,49]. In the conversion of monovalent to multivalent formats, the design of linkers that connect individual binding domains plays a critical role, as linker length and flexibility strongly influence the efficacy of multivalent engagement. Linker dynamics can determine whether simultaneous or sequential epitope binding is achieved, thereby modulating avidity and functional potency. For example, 2H10, a nanobody targeting the membrane-proximal external region (MPER) of HIV gp41, was engineered into bivalent formats using 15- or 17-residue glycine-serine (GS) linkers, resulting in enhanced HIV neutralization potency and breadth [56]. This case illustrates how a comparatively weak monovalent nanobody can be transformed into a substantially more potent inhibitor through rational valency and linker engineering. An additional strategy to enhance neutralizing activity involves the use of bispecific formats, in which two nanobodies targeting distinct viral epitopes are combined within a single construct. Using this approach, Matz et al. generated bispecific nanobodies by fusing JM2, JM3, JM4, and JM5, which target the CD4- and coreceptor-binding sites of HIV gp120 [89]. Notably, the JM5 × 2 and JM5 × 3 bispecific constructs outperformed their corresponding monovalent and bivalent formats in HIV neutralization assays [89].

Similarly, Fc-fusion proteins derived from anti-HIV nanobodies or small proteins, are typically bivalent, have been further engineered into multispecific formats to enable the simultaneous engagement of multiple epitopes, either on the same target or across distinct targets (Table 2). A notable example is sCD4, a domain that has been extensively characterized for its HIV neutralization potential but has not achieved clinical success in its monovalent form. Re-engineering sCD4 as an Fc-fusion protein and within multivalent bispecific IgG constructs has resulted in markedly enhanced HIV neutralization potency and breadth compared with the parent sCD4 domain [25]. Collectively, these studies underscore how the rational engineering of paratope, valency, linker architecture, and epitope specificity can yield protein-based inhibitors with substantially improved HIV neutralization potency and breadth.

## 4. Strategies to Extend Half-Life

Serum half-life is a critical consideration in the design of HIV entry inhibitors, as prolonged systemic exposure influences dosing frequency, durability of protection, and overall clinical feasibility [90]. Multiple strategies have been explored to improve the pharmacokinetic profiles of these inhibitors. One of the most straightforward approaches is the selection of an appropriate route of administration, which can substantially affect systemic exposure and persistence [31]. Studies have shown that intravenous, intramuscular, and subcutaneous administration generally provide more favorable pharmacokinetic profiles than the oral delivery [91,92,93]. Regardless of the administration route, protein-based inhibitors generally exhibit longer circulation times than small molecules or peptides owing to their larger size and reduced renal clearance [94]. Nevertheless, many engineered proteins, particularly those derived from non-human scaffolds, continue to exhibit suboptimal pharmacokinetics and may elicit immune responses [94]. Their rapid clearance can arise from inefficient engagement of endogenous recycling pathways, immunogenicity-driven elimination, or both [95].

To mitigate renal clearance, several chemical modification and fusion strategies have been developed [96]. PEGylation is one such strategy that increases hydrodynamic size and shields proteins from proteolytic degradation and immune recognition leading to extended serum half-life [97,98]. Though reports on improving serum half-life of HIV entry inhibitors by PEGylation are limited to some peptides and small molecules [99,100,101], the emergence of anti-PEG antibodies in clinical settings has raised concerns regarding the long-term utility of this approach [102,103]. This concern prompted the exploration of alternative fusion strategies that yield more humanized constructs. Accordingly, substantial efforts have focused on reducing immunogenicity while improving the serum half-life through humanization of protein-based HIV entry inhibitors, most commonly by fusing non-human scaffolds to human proteins or protein domains such as immunoglobulin (IgG) Fc domain, human serum albumin, or transferrin (Table 4).

Fusion of therapeutic proteins to the IgG Fc domain is one of the most widely used strategies for extending serum half-life. Although 14 Fc-fusion proteins have received FDA approval [104], none have yet been approved for HIV-1 entry inhibition. Fc fusion promotes interaction with the neonatal Fc receptor (FcRn), enabling FcRn-mediated recycling and protection from lysosomal degradation [105], thereby prolonging systemic exposure and reducing dosing frequency. Because IgG pharmacokinetics are largely governed by the pH-dependent interaction between Fc and FcRn, numerous Fc-engineering strategies have been developed to enhance FcRn binding under acidic endosomal conditions while preserving rapid dissociation at physiological pH. Well-characterized examples include the YTE (M252Y/S254T/T256E) [106] and LS (M428L/N434S) [107] variants, both of which substantially extend serum half-life in preclinical and clinical studies. More recently, the DHS (L309D/Q311H/N434S) variant was shown to function as a pH-responsive molecular switch that futher enhances circulation persistence [108]. Additional mutations, including RL (T307R/E380L) [109] and REW (T307R/E380W) [110], have also been improved FcRn-mediated recycling. However, the benefits of Fc engineering are context dependent, and the magnitude of half-life extension can vary among different antibodies and Fc-fusion constructs, necessitating empirical optimization for individual candidates.

While Fc fusion is highly effective for improving pharmacokinetics, it can also introduce Fc-mediated immune effector functions that may be undesirable in certain therapeutic applications. To address this limitation, several effector-silencing strategies have been developed. Early approaches focused on aglycosylation through mutations such as NG (N297G) [111,112] and DANG (D265A/N297G) [113], which disrupt the conserved N-linked glycosylation site at residue N297. Similarly, antibodies and Fc-fusion proteins produced in bacterial expression systems are aglycosylated and exhibit markedly reduced effector function [36]. However, aglycosylation alone does not completely eliminate Fc-mediated activity [114]. Consequently, additional Fc variants, including LALA (L234A/L235A) [115,116], LALA-PG (L234A/L235A/P329G) [117,118], FES (L234F/L235E/P331S) [119], and FQQ (L234F/L235Q/K322Q) [120], have been developed to more effectively silence effector functions. Several antibodies and Fc-fusion proteins containing these mutations have advanced to clinical evaluation. Importantly, combining pharmacokinetics-enhancing mutations with effector silencing mutations can generate molecules with both improved pharmacokinetic properties and minimized immune effector activity [120,121]. Beyond direct Fc fusion, conjugation of anti-HIV proteins to IgG Fc-binding peptides has also been shown to extend serum half-life [10]. Collectively, these advances highlight the considerable therapeutic potential of Fc-based engineering strategies for the development of long-acting HIV entry inhibitors.

In protein biotechnology, not all fusion constructs are easy to express. In such cases proteins such as albumin and transferrin can also be explored when only enhancement of serum half-life is desired. Fusion to human serum albumin [122] or incorporation of albumin-binding domains [123,124,125] or nanobodies [126] leverages the naturally long half-life of albumin, which is also maintained through FcRn-dependent recycling. This strategy has proven particularly effective for small protein scaffolds, such as peptides and nanobodies, where albumin binding can substantially improve pharmacokinetic profiles without significantly increasing molecular complexity or immunogenicity [49,127,128].

Another promising strategy is fusion to transferrin protein to enhance serum persistence. Transferrin is an abundant plasma protein with a relatively long half-life and undergoes receptor-mediated recycling via the transferrin receptor (TfR), allowing transferrin-fused proteins to evade rapid clearance [129,130]. In addition to extending circulation time, transferrin fusion has several advantages. It may facilitate targeted tissue distribution as TfR is widely expressed on proliferating and metabolically active cells. It can deliver drugs across blood-brain barrier [131,132] and can be absorbed orally effectively owing to the transferrin receptor transcytosis mechanism [133,134,135]. So far there is only one report of using this approach in the HIV field, namely, fusing an anti-transferrin antibody to the antisense peptide-nucleic acid specific for the *rev* gene of HIV-1 leading to a 15-fold improvement in delivery to the brain showing a promising role in neurologic AIDS [136]. Some other proteins that can be used to extend half-life are unstructured polypeptide like XTEN and carboxyl-terminal peptide that protect the fused protein from rapid breakdown and increase the hydrodynamic volume of the fused proteins keeping them well above the renal filtration threshold [95,137].

Collectively, these half-life extension approaches are central to optimizing the pharmacokinetic and dosing profiles of HIV entry inhibitors. Improving systemic exposure enables less frequent dosing, which may enhance patient adherence and support long-acting therapeutic or prophylactic applications. The recent emergence of long-acting antiretroviral agents, such as lenacapavir [138], has raised the benchmark for protein-based HIV entry inhibitors. To remain clinically competitive, these candidates will need to combine potent, broad antiviral activity with prolonged systemic exposure and infrequent dosing while maintaining durable efficacy. However, careful optimization is required to ensure that such strategies do not compromise target engagement or antiviral potency.

## 5. Strategies to Activate Host Immune Functions Against HIV Infection

While early antibody-based therapies primarily relied on direct viral neutralization, newer approaches, including Fc-fusion proteins, increasingly exploit Fc-mediated immune effector functions to enhance antiviral activity [139,140]. Fc-dependent mechanisms can make substantial contributions to therapeutic efficacy; for example, Fc effector functions account for approximately 21% of the anti-HIV activity of bnAb VRC07-523LS [141]. Engagement of Fc gamma receptors (FcγRs) on immune effector cells and complement component C1q promotes viral clearance through antibody-dependent cellular cytotoxicity (ADCC), antibody-dependent cellular phagocytosis (ADCP), antibody-dependent cellular trogocytosis (ADCT), and complement-dependent cytotoxicity (CDC) (Table 4) [142]. Evidence from both preclinical and clinical studies indicates that these mechanisms contribute to viral control by limiting HIV spread and facilitating the elimination of infected cells [36,141,143]. Although the native Fc domain can mediate these activities, Fc engineering has enabled the development of variants with enhanced binding to FcγRs and C1q, thereby amplifying immune effector functions and improving antiviral efficacy.

There are several approaches to Fc engineering. One strategy involves introducing specific amino acid substitutions within the Fc domain to increase affinity for activating FcγRs, including FcγRI, FcγRIIa, and FcγRIIIa, thereby enhancing ADCC and ADCP. For example, Fc variants containing the DE (S239D/I332E) or DLE (S239D/A330L/I332E) mutations exhibit improved engagement of FcγRIIIa and increased effector cell activation [144]. While CDC activity is maintained in the DE variant, it is reduced in the DLE variant [144]. These findings highlight the need for careful, rational mutagenesis to avoid compromising immune effector functions normally mediated by the wild-type Fc domain. More recently, RL (Q311R/M428L) and REW (Q311R/M428E/N434W) variants were reported to improve serum half-life while enhancing ADCC and CDC activities [109,110]. Conversely, selective attenuation of inhibitory FcγRIIb binding can further bias immune responses toward activating pathways. For example, the GAALIE (G236A/A330L/I332E) mutation markedly reduced FcγRIIb binding while selectively enhancing interactions with activating FcγRs, including FcγRIIa and FcγRIIIa [145]. Incorporation of such Fc-engineering strategies into anti-HIV Fc-fusion proteins may improve antiviral potency and clinical performance.

Glycoengineering of the Fc domain represents another powerful approach to enhance effector functions. Removal of core fucose (afucosylation) from Fc N-linked glycans markedly increases FcγRIIIa binding and ADCC potency, a strategy that has been successfully translated into approved therapeutic antibodies [146,147]. Afucosylation removes the steric hindrance offered by fucose, thus enhancing Fc-FcγRIIIa interaction [148]. Afucosylated Fc variants have also been explored in the context of HIV antibodies, where enhanced ADCC activity has been associated with improved anti-HIV efficacy [149,150,151].

Multivalency and multispecific antibody formats further amplify effector function by increasing immune synapse formation and promoting more efficient clustering of Fcγ receptors on effector cells [152]. Bispecific and trispecific antibodies that simultaneously engage multiple target epitopes or multiple receptors on immune cells, enhance both neutralization and Fc-mediated clearance [153,154]. Additionally, Fc fusion to non-antibody scaffolds, such as nanobodies or lectins confer effector functionality to otherwise Fc-silent molecules [32,36,40].

While enhancing effector functions holds promise for improving therapeutic efficacy, these strategies must be carefully balanced against safety considerations. Excessive FcγR activation or complement engagement may increase the risk of immunopathology, inflammation, or off-target effects [155,156]. Consequently, rational design of HIV entry inhibitors increasingly involves fine-tuning Fc-mediated effector functions to achieve optimal anti-HIV activity while maintaining an acceptable safety profile.

## 6. Strategies to Improve Manufacturability

While production of small proteins like nanobodies, lectins, and soluble domains of host receptors is comparatively easier, production of Fc-fusion proteins and antibodies can be tricky. Efficient and scalable production is a critical consideration in the clinical translation of protein-based HIV entry inhibitors. High manufacturing yields, robust product quality, and cost-effective processes are essential to support large-scale deployment of any HIV entry inhibitor with the potential for long-acting therapeutics and prophylactic applications. Consequently, significant efforts have been directed toward optimizing production and purification systems, molecular design, and downstream processing and formulation strategies to improve manufacturability and developability.

Selection of an appropriate production platform is a primary determinant of production efficiency. Mammalian expression systems, such as Chinese hamster ovary (CHO) [157,158], HEK293 [159], and Expi293 [160] cells, are widely used for production of antibodies and Fc-fusion proteins due to their ability to support proper folding, post-translational modifications, and human-like glycosylation profiles. Mammalian expression systems can be used to produce antibodies in two ways: one is via transient gene expression (TGE) and another is via stable gene expression (SGE). In SGE, a gene encoding antibody is permanently integrated into the host genome, which is a slow and labor-intensive process [161]. Though SGE is highly reproducible and consistent over time and suitable for large-scale biomanufacturing, relatively low yields are observed [161]. In TGE, on the other hand, a gene encoding antibody remains on the plasmid only and genome integration is not required for selection, making it a relatively fast process. Because multiple copies of the plasmid per cell are present, higher yields are obtained [161]. So far, TGE is the method of choice and has been optimized in several parameters to get the higher antibody yields [157,160,162]. For example, optimization of transfection conditions and growth medium was shown to enhance the production of recombinant Fc-fusion proteins and antibodies in TGE [159,163]. To improve the manufacturability, Hederoth et al. performed several optimizations to reduce the cost of production in Expi293 cells [163]. They replaced Expi293 expression medium with BalanCD HEK293 medium that reduced the medium costs by approximately 90% and they used salmon sperm DNA as filler DNA during transfection that minimized plasmid DNA usage [163]. Similarly, Heng et al. showed 4-fold and 2.5-fold improved production of light chain and whole antibody, respectively by systematically optimizing the transfection conditions, culture volume and temperature, and medium supplements [159]. DMSO and lithium acetate supplementation in transiently transfected CHO cells increased the antibody yield upto 80 mg/L [164]. There are several other factors that require optimization for enhanced antibody or Fc-fusion protein production such as vector and promoter engineering, use of signal peptides and regulatory elements, and co-expression of helper genes, which has been reviewed in detail by Fu et al. [102].

*Nicotiana benthamiana* and tobacco BY-2 suspension cell cultures have emerged as attractive alternatives to mammalian expression systems for the production of Fc-fusion proteins. Several anti-HIV Fc-fusion proteins have been successfully expressed in these systems and shown to retain binding to FcγRI and FcγRIIIa receptors. For example, production of the anti-HIV avaren-Fc (AvFc) in *N. benthamiana* achieved yields of approximately 100 mg/kg and demonstrated both HIV-neutralizing activity and Fc-mediated immune effector functions [40]. Similarly, Gutierrez-Valdez et al. produced anti-HIV CD4-Fc-C34 and CD4-Fc-T20 Fc fusion proteins in *N. benthamiana* and BY-2 cell suspension; however, efficacy remains to be evaluated, and production yields, currently reported at 4 mg/kg, require further optimization to support large scale manufacturing [165]. In addition, a bispecific construct generated by fusing VRC01_Fab_ to avaren was successfully produced in *N. benthamiana* and exhibited improved HIV-1 neutralization compared with an equimolar mixture of VRC01_Fab_ and avaren [41]. Collectively, these studies highlight the potential of plant-based expression platforms as an alternative approach for the production of Fc-fusion proteins.

Alternative expression systems, including yeast and microbial platforms, are attractive for producing less complex antiviral proteins such as nanobodies [9,32], lectins [36,37,63], and soluble receptor domains [166], because they combine rapid growth with lower production costs and high expression yields.

Purification is another key determinant of production efficiency. Recombinantly expressed proteins are commonly engineered with affinity tags, such as His_6_ or Strep tags, to facilitate purification. For Fc-fusion proteins, however, the Fc domain not only improves stability and pharmacokinetics but also enables purification through standard affinity chromatography workflows, eliminating the need for additional affinity tags. Commercial Protein A- and Protein G-based remain the industry standard for purifying antibodies and Fc-fusion proteins and continue to be optimized for higher throughput and compatibility with increasingly complex antibody formats [167]. For example, HiScreen Fibro PrismA columns reduce purification times by approximately 80% without compromising yield or purity through substantially higher operating flow rates (up to 30 mL/min), compared to traditional Protein-A columns (~5 mL/min) [163]. Alternative purification strategies are also emerging. For example, human acetylcholinesterase fused to an IgG1 Fc domain was recently purified using aromatic Zn^2+^ chelator complexes in the presence of polyethylene glycol, providing a potentially economical alternative to conventional affinity chromatography for Fc-fusion proteins [168].

Purification of multispecific antibody-based constructs presents additional challenges because chain mispairing, aggregation, and low-abundance product species complicate large-scale manufacturing. Knob-in-hole engineering remains one of the most widely adopted strategies for promoting correct heavy-chain heterodimerization [169] and numerous manufacturing and purification workflows have been developed around this platform [170,171,172]. Additional purification strategies further improve heterodimer recovery [171,172]. For instance, engineering asymmetric Protein A binding by weakening one Fc domain (Fc*) while retaining the other (Fc) enables chromatographic separation of heterodimers from homodimers based on differential Protein A affinity [173]. Likewise, the MabSelect family of resins has demonstrated improved purification performance for asymmetric bispecific antibody formats compared with conventional Protein A resins [174].

Collectively, advances in expression, purification and manufacturability are essential for translating protein-based HIV entry inhibitors from laboratory-scale discovery to clinical application. Integrating scalable manufacturing strategies with approaches that enhance potency and pharmacokinetics will be critical for advancing these inhibitors toward widespread clinical use.

## 7. Challenges in Preclinical and Clinical Translation

As discussed throughout this review, a diverse arsenal of protein-based HIV entry inhibitors has demonstrated potent antiviral activity in vitro, with some candidates also showing promising prophylactic and therapeutic efficacy in preclinical models. However, successful translation from in vitro activity to in vivo efficacy remains a major challenge in infectious disease drug development. Experimental systems cannot fully recapitulate the complexity of the host environment, where factors such as interactions or competition with host components, immunogenicity, pharmacokinetics, tissue distribution, protein stability, and differences in target accessibility or behavior in host system can substantially influence therapeutic performance [175]. Consequently, candidates exhibiting exceptional in vitro potency may show reduced or variable efficacy in vivo. Also, preclinical evaluation of HIV therapeutics presents its own set of challenges. First, the availability of suitable animal models is limited, and each model has inherent constraints. Rhesus macaques are among the most widely used and clinically relevant non-human primate models; however, because HIV-1 does not productively infect these animals, studies generally rely on simian-human immunodeficiency viruses (SHIVs) [176,177]. Although highly valuable, SHIV models do not fully recapitulate HIV-1 infection in humans, which can limit the direct translation of experimental outcomes. Moreover, non-human primate studies are costly, resource-intensive, and require specialized facilities and expertise. Preclinical studies also demand substantially larger quantities of therapeutic candidates than in vitro experiments, creating additional challenges related to protein production, purification, formulation, and scalability. More recently, humanized mouse models have emerged as a valuable alternative for evaluating HIV infection and therapeutic interventions in vivo [178]. However, these models are also relatively expensive, and variability in the efficiency of human immune-cell engraftment can contribute to substantial inter-animal variability. Careful selection and stratification of animals based on the extent of human hematopoietic reconstitution, commonly assessed by the percentage of human CD45^+^ cells, can help minimize this variability. Nevertheless, compared with non-human primate models, humanized mice offer the important advantage of requiring substantially smaller quantities of therapeutic candidates, making them particularly useful for the early preclinical evaluation of protein-based HIV entry inhibitors. A second major challenge is the complexity of conducting HIV infection studies, which involve exposure to infectious virus and therefore require specialized high-containment facilities, trained personnel, and extensive institutional and regulatory approvals. These requirements substantially increase the logistical burden, time, and cost associated with in vivo efficacy studies, further limiting the number of promising candidates that can undergo comprehensive preclinical evaluation. Even when suitable animal models and biosafety requirements are successfully addressed, candidates with suboptimal in vivo performance may require additional engineering and iterative testing. Improvements in potency, stability, or pharmacokinetics may also introduce trade-offs in protein expression, aggregation, or manufacturability, requiring iterative rounds of engineering and in vivo validation. Collectively, these biological and logistical constraints limit the number of promising protein-based HIV entry inhibitors that can be comprehensively evaluated preclinically.

Despite these hurdles, several protein-based HIV entry inhibitors have progressed to preclinical evaluation, with many demonstrating encouraging efficacy in vivo (Table 3). Their subsequent transition into clinical trials, however, remains limited. Clinical advancement requires not only favorable preclinical efficacy, safety, and tolerability, but also substantial financial and regulatory support, suitable study populations, and robust manufacturability. Key candidates must be amenable to reproducible, scalable production and purification, exhibit high solubility and stability, have a low propensity for aggregation, and desirable formulation characteristics. Most HIV biologics currently advancing through clinical development are bnAbs, supported by their established antiviral efficacy, favorable safety profiles, and relatively low immunogenicity [12]. In contrast, alternative protein-based entry inhibitors, such as lectins and nanobodies, may require closer consideration of immunogenicity, particularly when derived from non-human sources, which may necessitate further engineering or humanization to improve their clinical suitability. Collectively, these considerations narrow the pool of candidates suitable for advancement to clinical development.

## 8. Conclusions and Future Perspectives

The current therapeutic landscape for HIV is dominated by combination antiretroviral regimens targeting reverse transcriptase, integrase, and protease, with integrase strand transfer inhibitor (INSTI)-based regimens recommended as first-line therapy for most people living with HIV [179]. Although these agents remain the cornerstone of effective HIV management, the emergence of drug resistance, treatment-related limitations, and the need for lifelong therapy underscore the importance of continued therapeutic innovation. Therefore, sustained efforts to develop alternative strategies with distinct mechanisms of action are essential to broaden the therapeutic arsenal and provide effective options when existing treatments become inadequate or ineffective.

Targeting HIV entry offers several advantages over therapies that inhibit intracellular stages of the viral life cycle. Because these agents act extracellularly, they circumvent the need for cellular uptake and can block HIV infection before viral entry, unlike reverse transcriptase, integrase, or protease inhibitors, which act after entry. Moreover, certain classes of entry inhibitors, including Fc-fusion proteins and monoclonal antibodies, provide an additional therapeutic advantage by engaging immune effector mechanisms that promote the clearance of HIV-infected cells. By offering distinct mechanisms of action from those of conventional antiretroviral drugs, they provide attractive complementary or alternative options in the context of treatment resistance.

As highlighted throughout this review, protein-based entry inhibitors, including nanobodies, lectins, soluble receptor derivatives, and engineered antibodies, have demonstrated remarkable anti-HIV activity in vitro (Table 2). However, relatively few non-bnAb protein-based entry inhibitors have been evaluated in vivo (Table 3), and clinical advancement of these alternative platforms remains limited. Addressing key translational challenges, including pharmacokinetics, immunogenicity, manufacturability, and in vivo efficacy will be essential for realizing their therapeutic potential.

**Figure 2:**
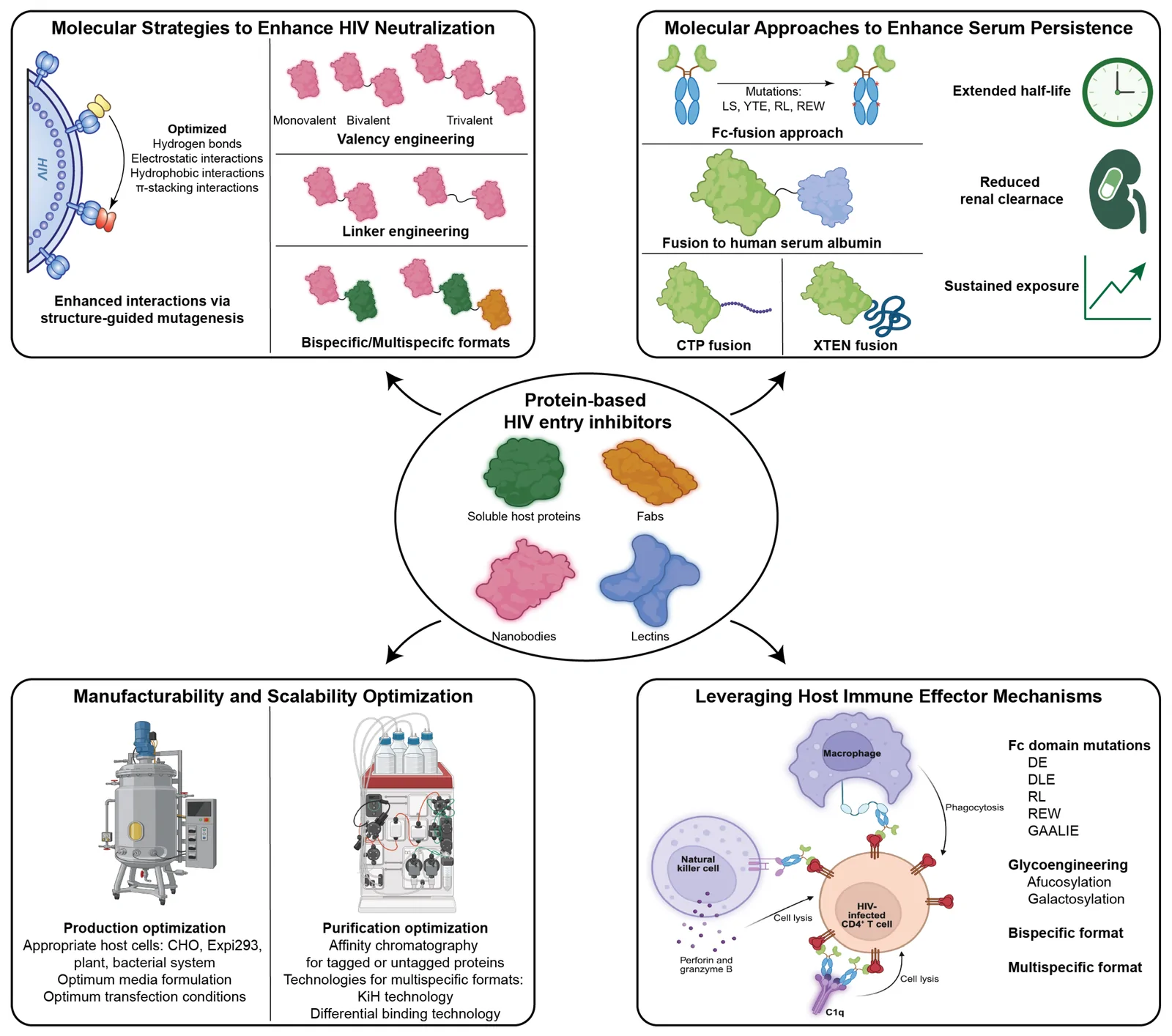
Strategies for developing next-generation HIV entry inhibitors with enhanced antiviral potency, durability, and developability. Antiviral potency can be improved through engineering approaches that enhance HIV neutralization breadth and potency, as well as by harnessing host immune effector mechanisms to promote the elimination of infected cells. Durability may be achieved by extending serum half-life through fusion with IgG1 Fc domains, serum albumin, C-terminal peptide (CTP), or XTEN polypeptides. Developability can be optimized through the selection of appropriate expression hosts, transfection and production conditions, and purification strategies to maximize protein yield, stability, and manufacturability.

Numerous protein-engineering and Fc-engineering strategies have been successfully developed to enhance the pharmacokinetics, antiviral potency, manufacturability, and immune effector functions of therapeutic proteins (Figure 2). Despite their established utility in other therapeutic areas, these approaches remain underutilized in the development of HIV-1 entry inhibitors. Continued progress will require close integration of protein engineering technologies, structural biology, immunology and translational research to improve the therapeutic performance of existing candidates and accelerate their progression toward clinical evaluation. Protein-based HIV entry inhibitors could serve as independent interventions or complement existing approaches. For instance, in prevention they may provide sustained systemic or mucosal protection alongside current pre-exposure prophylactic strategies. Therapeutically, their combination with ART or bnAbs could enhance antiviral efficacy and increase the barrier to resistance. However, rigorous in vivo evaluation followed by well-designed clinical studies will be essential to translate promising nanobodies, lectins, Fc-fusion proteins, and other engineered protein scaffolds into practical interventions for HIV prevention and treatment. Collectively, continued innovation in protein design, coupled with rigorous translational studies, could establish protein-based HIV-1 entry inhibitors as an important component of the expanding arsenal of HIV prevention and treatment.

## Figures and Tables

**Table 1:** FDA-approved HIV-entry inhibitors [3].

Generic Name	Abbreviation and Other Name	Brand Name	FDA Approval Year	Category	Function	Target
Enfuvirtide *	T-20	Fuzeon	2003	Peptide	Fusion inhibitor	HIV-1 gp41
Maraviroc	MVC	Selzentry	2007	Small molecule	CCR5 antagonist	CCR5 coreceptor
Fostemsavir	Fostemsavir tromethamine, FTR	Rukobia	2020	Small molecule	Attachment inhibitor	HIV-1 gp120
Ibalizumab	IBA, Hu5A8, TMB-355, TNX-355	Trogarzo	2018	Monoclonal antibody	Post-attachment inhibitor	CD4 receptor

* Discontinued due to evolving treatment practices and reduced clinical need.

**Table 3:** Protein-based HIV entry inhibitors with proven in vivo anti-HIV efficacy.

Proteins	Engineered	Preclinical Studies				References
		Animal Model	HIV Strain	Dose	Efficacy	
sCD4	eCD4-Ig	Rhesus macaques	SHIV-AD8	2.5 × 10^13^ AAV1-encapsidated particles delivering AAV-rh-eCD4-Ig	Effective (Prophylaxis)	[29]
Nb_457_	Nb_457_-Nb_HSA_-Nb_457_	NDG-HuPBL mice	HIV-1_CH058_	20 mg/kg	Effective (Therapeutic)	[31]
	Nb_457_-Fc	NDG-HuPBL mice	HIV-1_CH058_	20 mg/kg	Ineffective (Therapeutic)	
GRFT	-	CD34^+^ NSG mice	HIV-1_BAL_	250 nmoles/kg	Effective (Prophylaxis)	[36]
	mGRFT-Fc	CD34^+^ NSG mice	HIV-1_BAL_	250 nmoles/kg	Effective (Prophylaxis)	
CV-N	-	*Macaca fascicularis*	SHIV89.6	1% or 2%	Effective (Topical microbicide)	[38]

**Table 4:** Fusion strategies applied to protein-based HIV entry inhibitors for improved serum-half-life and immune effector functions.

Strategies	Fusion Products	Fused Domains	Mutations	Serum Half-Life			Immune Effector Functions	References
				Animal Model	Route of Administration	Half-Life		
Fc fusion	mGRFT-Fc	IgG1-Fc domain	LS	Sprague-Dawley rats	Intravenous	30.4 h	ADCC and CDC	[36]
	Avaren-Fc	IgG1-Fc domain	None	Rats	Intravenous	18.8 h	ADCVI	[40]
				Rhesus macaques	Intravenous	27.7 h		
	Nb_457_-Fc	IgG1-Fc domain	None	NDG-huPBL mice	Intraperitoneal	7.35 h	nd	[31]
					Subcutaneous	10.93 h		
	1F10-Fc	IgG1-Fc domain	None	-	-	-	ADCP and ADCT	[32]
	2E7-Fc							
	J3-Fc							
	J3-1F10-Fc							
	J3-2E7-Fc							
HSA fusion	Nb_457_-Nb_HSA_-Nb_457_	HSA-binding nanobody	None	NDG-huPBL mice	Intraperitoneal	7.45 h	-	[31]
					Subcutaneous	27.71 h		

## Data Availability

No new data were created or analyzed in this study. Data sharing is not applicable to this article.
